# Proapoptotic and survival signaling in the neuroretina at early stages of diabetic retinopathy

**Published:** 2013-01-07

**Authors:** Angela M. Valverde, Soledad Miranda, Marta García-Ramírez, Águeda González-Rodriguez, Cristina Hernández, Rafael Simó

**Affiliations:** 1 Instituto de Investigaciones Biomédicas Alberto Sols (CSIC/UAM), Madrid, Spain; 2 Centro de Investigación Biomédica en Red de Diabetes y Enfermedades Metabólicas Asociadas (CIBERDEM), ISCIII, Spain; 3 Diabetes and Metabolism Research Unit. Vall d’Hebron Research Institute.Universitat Autònoma de Barcelona, Spain

## Abstract

**Purpose:**

Diabetic retinopathy (DR) has been classically considered a microcirculatory disease of the retina. However, before any microcirculatory abnormalities can be detected in ophthalmoscopic examination, retinal neurodegeneration is already present. The aim of the study was to analyze proapoptotic and survival signaling in the neuroretinas of diabetic patients at early stages of DR.

**Methods:**

The retinas from five diabetic donors at early stages of DR were compared with the retinas from five nondiabetic donors matched by age. Glial activation was evaluated by assessing glial fibrillar acidic protein (GFAP) with western blot and immunofluorescence. Proapoptotic molecules (FasL, procaspase-8, active caspase-8, total Bid, truncated Bid, Bim, and active caspase-3), as well as antiapoptotic markers (FLIP, BclxL, and cyclooxygenase-2 [COX-2]) were assessed with western blot.

**Results:**

GFAP and proapoptotic molecules (FasL, active caspase-8, truncated Bid (t-Bid), Bim, and active caspase-3) were significantly increased in the neuroretinas from diabetic patients compared to the control neuroretinas. In contrast, no significant differences in the expression of the antiapoptotic markers were found.

**Conclusions:**

An imbalance between proapoptotic and survival signaling was found in diabetic neuroretinas. Our results reveal key mechanistic pathways involved in the neurodegenerative process that occurs in the early stages of DR.

## Introduction

Diabetic retinopathy (DR) remains the leading cause of blindness among working-age individuals in developed countries. Current treatments for DR are indicated in too advanced stages of the disease and are associated with significant adverse effects. Therefore, new pharmacological treatments for the early stages are needed.

DR has been classically considered a microcirculatory disease of the retina. However, increasing evidence suggests that retinal neurodegeneration is an early event in the pathogenesis of DR that predates and participates in the microcirculatory abnormalities that occur in DR [1-6]. In fact, we have found the main features of retinal neurodegeneration (apoptosis and glial activation) in retinas from diabetic donors with mild or even without any microcirculatory abnormality appearing in ophthalmologic examinations performed during the year before death [7-9].

Diabetes increases apoptosis in neurons, especially in the inner retina, where retinal ganglion cells (RGCs) are located [6]. This loss of neural cells results in a reduction in the thickness of the retinal nerve fiber layer, which has been detected in rats with streptozotocin (STZ) diabetes and in clinical studies using scanning laser polarimetry [10] or optical coherence tomography [11,12]. This thinning of the ganglion cell layer has also been found in diabetic patients without or with only minimal DR [11-13]. In several experimental models of diabetic retinopathy, activation of death receptors and mitochondrial damage by oxidative and endoplasmic reticulum stressors are major triggers of apoptosis that ultimately lead to cellular damage [14-17]. However, little is known regarding the activation of these signaling pathways in the neuroretinas of diabetic patients. Research on the molecular mechanisms involved in apoptosis of the neuroretina could facilitate the design of new therapies aimed at preventing or ameliorating the progression of DR at early stages. Accordingly, in the present study we evaluated key molecules that regulate proapoptotic and survival signaling in the neuroretinas of diabetic patients in the early stages of DR.

## Methods

### Human samples

Five human post-mortem eyes were obtained from five consecutive type 2 diabetic donors between March 2011 and January 2012. All ocular tissues were used in accordance with applicable laws and with the Declaration of Helsinki for research involving human tissue. In addition, this study was approved by the ethics committee of our hospital. The mean duration of diabetes was 8.1±3.2 years, and all patients had mild non-proliferative DR in ophthalmologic examinations performed during the preceding two years. The causes of death were cardiovascular disease (n=4) and malignant neoplasm (n=1).

Five eyecups obtained from nondiabetic donors closely matched by age (69.1±7.4 versus 68.3±6.5 years) were selected from our eye bank as the control group. In both groups, the time elapsed from death to eye enucleation was less than 4 h. After enucleation, the eyes were snap frozen at –80 °C and stored until assayed. The neuroretina and the retinal pigment epithelium (RPE) were harvested under the microscopic dissection of isolated eyecups from donors. Three retinal sections of each retina were obtained from the central area (around the optic nerve).

### Protein extraction from human neuroretina

Protein extracts and tissue sections from samples of neuroretinas from diabetic and control individuals were prepared. Protein extracts from the neuroretina were prepared by homogenization with lysis buffer containing 50 mM Tris-HCl, 1% Triton X-100, 2 mM ethylene glycol-bis (beta-aminoethyl ether)-N,N,N',N'-tetra acetic acid( EGTA), 10 mM EDTA acid, 100 mM NaF, 1 mM Na_4_P_2_O_7_, 2 mM Na_3_VO_4_, 100 μg/ml phenylmethylsulfonyl fluoride, 1 μg/ml aprotinin, 1 μg/ml pepstatin A, and 1 μg/ml leupeptin. using the Brinkman PT 10/35 Polytron (ALT, East Lyme, CT). Extracts were kept ice-cold at all times. Extracts were cleared by microcentrifugation at 40,000 ×g for 20 min at 4 °C. The supernatant was aliquoted and stored at −70 °C. All ocular tissues were used in accordance with applicable laws and with the Declaration of Helsinki for research involving human tissue. In addition, this study was approved by the ethics committee of the Institut de Recerca Hospital Universitari Vall d’Hebron (Barcelona, Spain).

### Western blotting

After sodium dodecyl sulfate polyacrylamide gel electrophoresis (SDS–PAGE) was performed, gels were transferred to Immobilon membranes (Millipore) and were blocked using 5% non-fat dried milk or 3% bovine serum albumin in 10 mmol/l Tris-HCl and 150 mmol/l NaCl pH 7.5, and incubated overnight with several antibodies in 0.05% Tween-20, 10 mmol/l Tris-HCl, and 150 mmol/l NaCl pH 7.5. All primary antibodies were used at 1:1000 dilutions. Immunoreactive bands were visualized using the enhanced chemiluminescence (ECL) western blotting protocol (Millipore). Anti-Bim (Ref. 559685) and anti- BclxL (Ref. 610211) antibodies were from BD Biosciences PharMingen (San Diego, CA). Anti-Bid (Ref. AF860) and anti-FLIP (Ref. AF821) antibodies were from R&D Systems. Anticleaved (Asp175) caspase-3 antibody (#9661) was purchased from Cell Signaling (Beverly, MA). Anticaspase-8 (sc-7890) and anticyclooxygenase-2 (anti-COX-2; sc-19.999) antibodies were from Santa Cruz Biotechnology (Palo Alto, CA). Anti-FasL antibody (AB-16982) was from Upstate (Millipore). The assessment of glial fibrillar acidic protein (GFAP) with western blot (GFAP antibody: Ab7260, Abcam, Cambridge, UK) has been also described elsewhere [7]. Briefly, a total of 5 μg protein from the neuroretina was resolved by 12% SDS-PAGE and transferred to a polyvinylidene fluoride membrane (Millipore, Billerica, MA). The membranes were incubated with a primary antibody against human glial fibrillar acidic protein (GFAP), diluted 1:200,000, and further incubated with peroxidase-conjugated secondary antibody (Bio-Rad Laboratories). Proteins were visualized using the enhanced chemiluminescence detection system (Supersignal CL-HRP Substrate System; Pierce, Rockford, IL). The same blot was stripped and reblotted with an antibody specific to β-actin (Santa Cruz Biotechnology, Santa Cruz, CA) to normalize the GFAP levels. For densitometric analysis of western blots, Image J software was used.

### Glial fibrillar acidic protein immunofluorescence

GFAP immunofluorescence in the neuroretina was performed as previously described [9]. Briefly, tissue sections were incubated overnight at 4 °C with the primary antibody chicken antihuman GFAP (1:1000; Abcam). After washing, sections were incubated with Alexa Fluor 568 (Molecular Probes; Eugene, OR) secondary antibody for 1 h. GFAP immunofluorescence in the neuroretina was quantified using laser confocal microscopy (FluoView ASW 1.4; Olympus, Hamburg, Germany), and the units represent the total fluorescence intensity values corresponding to ten field images (40X NA:0.9) of each retina sample.

### Transferase-mediated dUTP nick-end labeling

Apoptosis was analyzed with the In situ Cell Death Detection Kit with fluorescein isothiocyanate (FITC; Roche, Barcelona, Spain), which is based on transferase-mediated dUTP nick-end labeling reaction following the manufacturer’s instructions. In summary, paraffin-embedded eye sections (7 μm thickness) from diabetic and nondiabetic donors were deparaffinized with xylene and rehydrated in ethanol. Then, a 50 μl terminal deoxynucleotidyl transferase dUTP nick end labeling (TUNEL) reaction mixture was added to each section and incubated in a humidified atmosphere for 60 min at 37 °C in the dark. Images were acquired in a confocal laser scanning microscope (FV1000, Olympus) using either a 488 nm laser line for FITC or a 405 nm laser line for 4',6-diamidino-2-phenylindole (DAPI). Each retinal section was visually scanned with a high-power lens (60X) that covered 212 × 212 μm of each retina. The total number of TUNEL-positive cells was recorded in a masked fashion using the values corresponding to 15 field frame images from three retinal sections (five fields each). The mean value was then standardized (mathematically converted) to a 0.5-cm^2^ area. To avoid false-positive fluorescence, a phase-contrast image for each field was also obtained.

### Statistical analysis

Results are means±SD. Statistical significance was tested with a one-way analysis of variance followed by the protected least-significant difference test; p<0.05 was considered significant. In experiments using X-ray films, different exposure times were used to ensure that bands were not saturated.

## Results

### Biomarkers of retinal neurodegeneration

**Figure 1:**
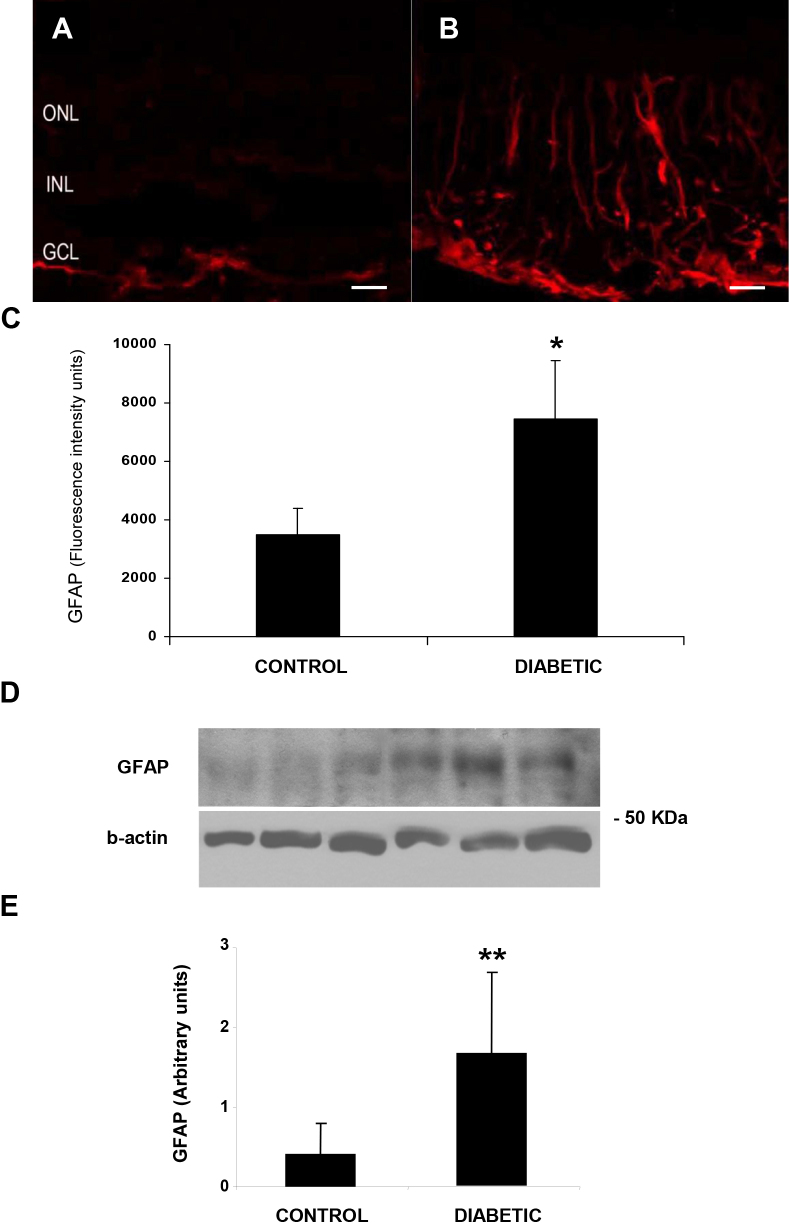
Glial activation in neuroretina from diabetic patients. Comparison of glial fibrilllar acidic protein (GFAP) immunofluorescence (red) in the human retina between representative samples from a nondiabetic (**A**) and diabetic (**B**) donor. In the diabetic retina, the Müller cells show abundant GFAP immunofluorescence and the radial processes stain intensely throughout the inner and outer retina. ONL, outer nuclear layer; INL, inner nuclear layer; GCL, ganglion cell layer. The bar represents 20 μm. **C**: Quantification of GFAP immunofluorescence in nondiabetic (n=5) and diabetic retinas (n=5). **D**: Western blot analysis of neuroretinas corresponding to three representative diabetic and nondiabetic retinas. **E**: Densitometric quantification showing higher GFAP in the retinas from five diabetic donors compared with the retinas from five nondiabetic donors. Results are expressed as means±SD. *p<0.001. **p=0.04.

As expected, the two main features of retinal neurodegeneration (apoptosis and glial activation) were present in the diabetic retinas. A higher intensity of GFAP immunofluorescence was detected in the diabetic retinas (7,450±927 versus 3,473±819, p<0.001; Figure 1A-B). In addition, an increase in GFAP assessed with western blot (arbitrary units) was observed in the neuroretinas from diabetic patients compared with nondiabetic individuals (1.64±1.03 versus 0.40±0.39, p=0.04; Figure 1C-D). Furthermore, the diabetic retinas presented a higher ratio of apoptosis (percentage of TUNEL positive cells per 0.5 cm^2^) than nondiabetic retinas (21±9 versus 3±1; p<0.001).

### Differential proapoptotic and survival signaling in neuroretinas from diabetic patients and control individuals

**Figure 2:**
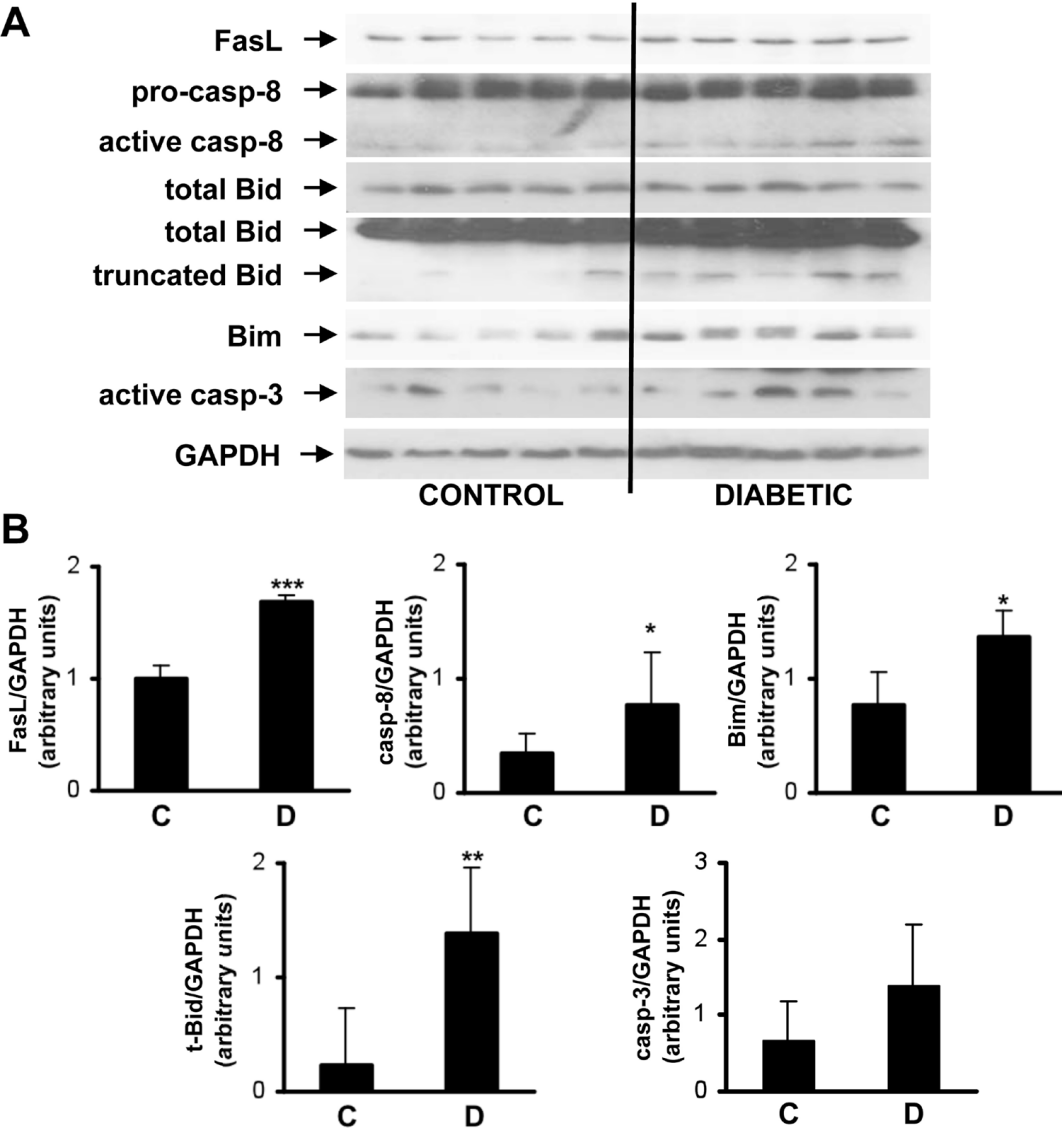
Apoptotic signaling pathways in neuroretina from diabetic patients. **A**: Protein extracts were prepared from neuroretina from diabetic patients (n=5) and nondiabetic control individuals (n=5). Total protein (50 μg) was used for western blot analysis with the antibodies against Fas ligand (FasL), caspase-8, BH3 interacting-domain death agonist (Bid), B-cell lymphoma 2 interacting mediator of cell death (Bim), and active caspase-3. Anti-GADPH antibody was used as a loading control. **B**: Autoradiograms were quantified with scanning densitometry. The results are expressed as arbitrary units of protein expression and are means±SD. *p<0.05, **p<0.01 and ***p<0.005 diabetic patients (**D**) vsersus control individuals (**C**).

**Figure 3:**
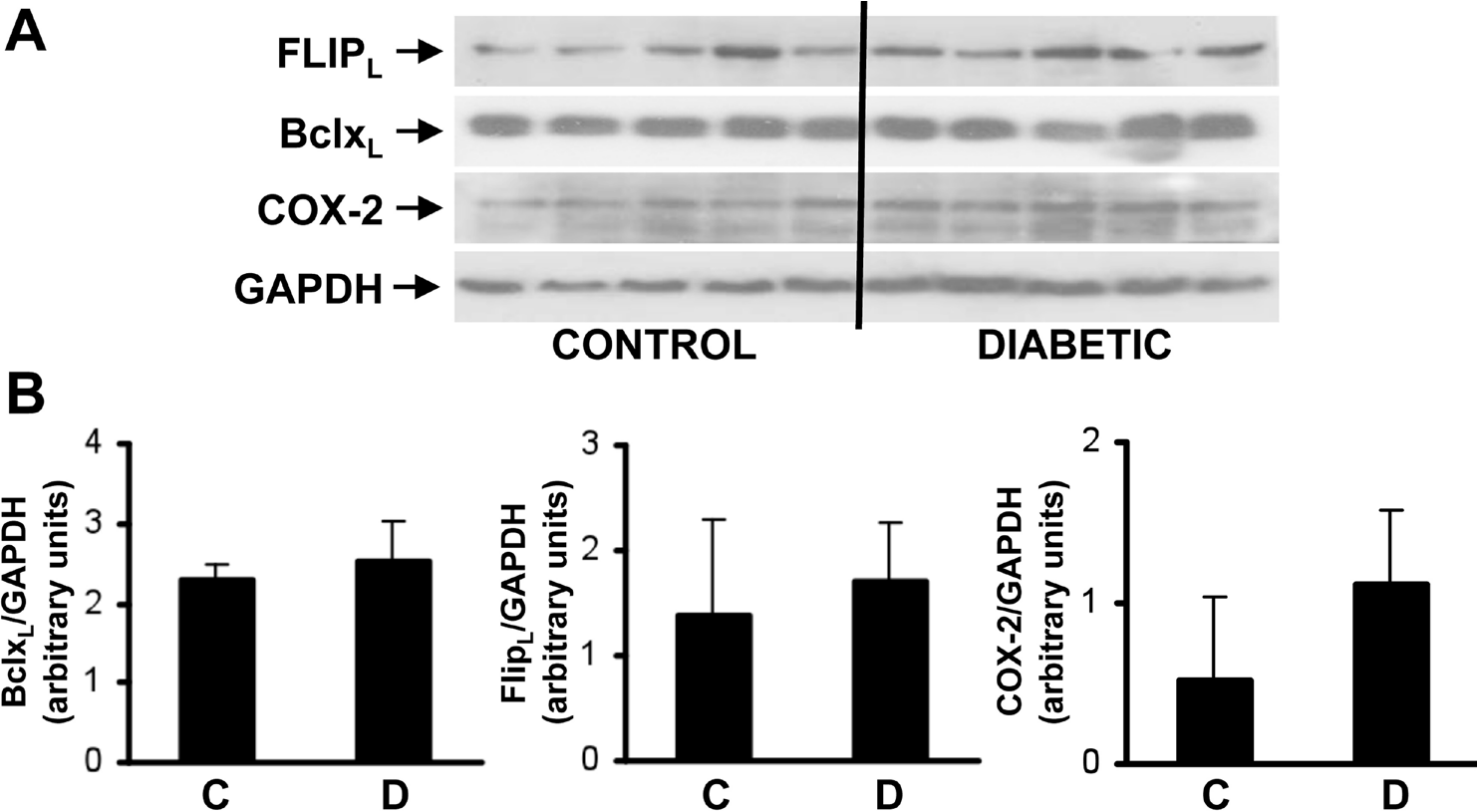
Survival signaling pathways in neuroretina from diabetic patients. **A**: Protein extracts were prepared from neuroretina from diabetic patients (n=5) and nondiabetic control individuals (n=5). Total protein (50 μg) was used for western blot analysis with the antibodies against B-cell lymphoma-extra large (BclxL), FLICE-like inhibitory protein (FLIP) and ciclooxygenase-2 (COX2). Anti-GADPH antibody was used as a loading control. **B**: Autoradiograms were quantified with scanning densitometry. Results are expressed as arbitrary units of protein expression and are means±SD. **C**: nondiabetic donors, **D**: diabetic donors.

The results of the proapoptotic and survival molecules measured in the retina from diabetic patients and nondiabetic control individuals are shown in Figure 2 and Figure 3. The expression of FasL, a proapoptotic component of the death receptor pathway, was significantly increased in the neuroretinas from diabetic patients compared to the control individuals. The interaction of FasL with the death receptor Fas/CD95 assembles the death-inducing signaling complex (DISC) [18]. This includes the recruitment of caspase-8. Activation of caspase-8, monitored by the presence of its 20 kDa active fragment, was significantly increased in neuroretinas from diabetic patients compared to the control individuals. These data were reinforced by the detection of elevated levels of truncated Bid fragment (tBid) in diabetic samples. Moreover, the proapoptotic Bcl2 family member Bim, which is involved in the mitochondria-mediated intrinsic apoptotic pathway, significantly increased in diabetic patients compared to the control individuals. As a result, the neuroretinas from diabetic patients displayed significant activation of the executer caspase-3, monitored by the presence of its 15–17 kDa active fragment, compared to the control individuals (Figure 2). In contrast, no significant changes in the expression of antiapoptotic markers such as FLIP, a component of the DISC, and BclxL, an antiapoptotic member of the Bcl2 family, were found between the diabetic and control individuals although a trend toward an increase in COX-2 content in diabetic patients was observed (Figure 3).

## Discussion

In the present study, we explored the proapoptotic signaling pathways involved in the neurodegenerative process that occurs in the early stages of DR in diabetic patients. As occurs in other cells, the imbalance between the proapoptotic and survival intracellular signaling pathways determines the extent of cellular damage in the neuroretina and, consequently, is critical for the pathogenesis of DR [19]. Therefore, studying these pathways will permit us not only to define the molecules that are altered by the diabetic milieu but also to gain new insights into the design of therapeutic strategies.

We recently reported that stress and survival signaling are oppositely regulated in retinal pigment epithelia from diabetic patients [20], but there is no information regarding this issue for the neuroretina. In this study, we demonstrated for the first time that activation of the death receptor pathway, monitored by the elevation in the expression of FasL and the activation of caspase-8, is significantly higher in neuroretinas from diabetic patients than in nondiabetic control individuals. Importantly, our data clearly show increased Bid cleavage in the neuroretinas from diabetic patients. This result suggests that in neuroretinas from diabetic patients the apoptotic signals emerging from the death receptor pathway are amplified to additional activation of the intrinsic apoptotic signaling pathway, resulting in stronger activation of the executer caspase-3. In conjunction, our results clearly demonstrate that targeting the activation of the Fas/FasL death receptor pathway might be a novel therapeutic strategy against DR. In light of our data, Fas was upregulated in rat neutrophils from streptozotocin-treated rats in parallel with a simultaneous increase in Fas expression in the retinal vasculature, thereby resulting in apoptosis in endothelial cells [21]. Moreover, Al-Asrar et al. reported a histological analysis of human diabetic retinas showing Fas and FasL immunoreactivity in ganglion and glial cells, respectively [22]. However, the latter report did not delineate the sequential activation of the molecular mediators of the Fas/FasL system in diabetic retinas as shown in the present study. Importantly, researchers recently reported that in addition to exerting death-promoting effects, the death receptor Fas can induce the secretion of inflammatory cytokines such as interleukin-6 (IL-6), IL-1β, IL-1α, IL-8, and monocyte chemotactic protein-1 (MCP-1) in different cell lines [23,24]. Thus, in addition to the activation of apoptosis, FasL might trigger additional inflammatory damage, and DR might progress, in part, as the result from chronic exposure of the neuroretina to proinflammatory cytokines. Additional research will unravel this relevant issue.

In contrast, no differences in the expression of antiapoptotic proteins were found between the two groups although a trend toward an increase in COX-2 expression was observed in the neuroretinas from diabetic patients, probably reflecting an antiapoptotic defense. Notably, all these findings were observed at early stages of DR.

In conclusion, an imbalance between proapoptotic and survival signaling was found in the neuroretinas of diabetic patients. Our results reveal key mechanistic pathways involved in the neurodegenerative process that occurs in the early stages of DR. The relevance of these findings should be interpreted in the context of identifying apoptotic markers that might be useful for designing new therapeutic strategies aimed at preventing the progression of DR.
